# Supplementary material for: Identification and functional analysis of non-coding regulatory small RNA FenSr3 in Bacillus amyloliquefaciens LPB-18
Source: PeerJ. 2023 May 15;11:e15236. doi: 10.7717/peerj.15236 (PMC10194069; doi:10.7717/peerj.15236)
Supplement: Supplemental Information 4 [file peerj-11-15236-s004.zip › KO/CK-vs-T1_map/map04550.html]

KEGG PATHWAY: Signaling pathways regulating pluripotency of stem cells - Reference pathway


|  |  |
| --- | --- |
| **Signaling pathways regulating pluripotency of stem cells - Reference pathway** |  |

[
Pathway menu
| Organism menu
| Pathway entry
| Show description
| User data mapping
]

Pluripotent stem cells (PSCs) are basic cells with an indefinite self-renewal capacity and the potential to generate all the cell types of the three germinal layers. The types of PSCs known to date include embryonic stem (ES) and induced pluripotent stem (iPS) cells. ES cells are derived from the inner cell mass (ICM) of blastocyst-stage embryos. iPS cells are generated by reprogramming somatic cells back to pluripotent state with defined reprogramming factors, Oct4, Sox2, Klf4 and c-Myc (also known as Yamanaka factors). PSCs including ES cells and iPS cells are categorized into two groups by their morphology, gene expression profile and external signal dependence. Conventional mouse-type ES/iPS cells are called 'naive state' cells. They are mainly maintained under the control of LIF and BMP signaling. On the other hand, human-type ES/iPS cells, which are in need of Activin and FGF signaling, are termed 'primed state'. However, these signaling pathways converge towards the activation of a core transcriptional network that is similar in both groups and involves OCt4, Nanog and Sox2. The three transcription factors and their downstream target genes coordinately promote self-renewal and pluripotency.

|  |  |  |
| --- | --- | --- |
| Reference pathway | 184% 150% 122% 100% 82% 67% 55% | 图片下载 |
